# EVALUATION OF UPPER GASTROINTESTINAL ENDOSCOPY IN PATIENTS UNDERGOING BARIATRIC SURGERY

**DOI:** 10.1590/S0102-6720201500S100012

**Published:** 2015-12

**Authors:** Maurício Saab ASSEF, Tiago Torres MELO, Osvaldo ARAKI, Fábio MARIONI

**Affiliations:** Service of Endoscopy, Department of Surgery, Santa Casa de São Paulo São Paulo, SP, Brazil

**Keywords:** Obesity, Bariatric surgery, Endoscopy

## Abstract

***Background*:**

Obesity has become epidemic, and is associated with greater morbidity and mortality. Treatment is multidisciplinary. Surgical treatment is a consistent resource in severe obesity. The indication of preoperative upper gastrointestinal endoscopy in asymptomatic patients is controversial; however, most studies recommend its implementation in all patients.

***Aim*:**

To analyze endoscopic performance in patients who were in preoperative for bariatric surgery and compare them with control group.

***Method*:**

A series of 35 obese patients in preoperative period for bariatric surgery compared with a control group of 30 patients submitted to upper endoscopy. There were analyzed clinical and endoscopic data.

***Results*:**

The mean age of the group of patients was 43.54 years. Most individuals in the group of patients were female with median BMI of 47.26kg/m^2^and in control group 24.21 kg/m^2^. The majority of patients were asymptomatic. Upper endoscopy was altered in 81.25% of asymptomatic patients. Endoscopic findings in the patient group were 57.1% resulting from peptic ulcer disease and 34.3% associated with GERD. The analysis of endoscopic findings in patients showed no significant difference in relation of the control group. The prevalence of *H. pylori* infection was 60% in patients.

***Conclusion*:**

It is recommended that the upper endoscopy should be made in all patients in the preoperative bariatric surgery period, although the degree of obesity is not related to a greater number of endoscopic findings. Obese patients do not have more endoscopic findings that non-obese individuals.

## Abstract (pt)

***Racional*:**

A obesidade tornou-se epidemia e está associada à maior morbimortalidade. Seu tratamento é multidisciplinar. O tratamento cirúrgico é recurso consistente nos casos de obesidade grave. A indicação da endoscopia digestiva alta pré-operatória em pacientes assintomáticos é controversa; no entanto, a maioria dos estudos recomenda sua realização em todos os pacientes.

***Objetivo*:**

Analisar os resultados da endoscopia em pacientes que se encontravam em pré-operatório de cirurgia bariátrica, e compará-los aos achados do grupo controle.

***Método*:**

Série de 35 pacientes obesos em pré-operatório de cirurgia bariátrica com um grupo controle de 30 pacientes submetidos à endoscopia no período de fevereiro a julho de 2014. Foram analisados dados clínicos e endoscópicos.

A média de idade do grupo de pacientes foi de 43,54 anos e a maioria deste grupo era do sexo feminino. A média do valor do IMC no grupo de pacientes foi de 47,26 kg/m2 e no grupo controle de 24,21 kg/m^2^. A maioria dos pacientes era assintomática. A endoscopia estava alterada em 81,25% dos pacientes assintomáticos. As alterações endoscópicas no grupo de pacientes foram 57,1% decorrentes da doença ulceropéptica e 34,3% associadas à DRGE. A análise do número de achados endoscópicos no grupo de pacientes em relação ao grupo controle não demonstrou diferença estatística significante. A prevalência da infecção do *H. pylori* foi de 60% no grupo de pacientes.

***Conclusão*:**

Recomenda-se que a endoscopia digestiva alta deva ser realizada em todos pacientes em pré-operatório de cirurgia bariátrica, embora o grau de obesidade não tenha relação com maiores alterações endoscópicas. Os obesos não têm maior número de alterações endoscópicas que os não obesos.

## INTRODUCTION

Obesity has become epidemic and is associated with increased morbidity secondary to various factors (comorbidities), including gastroesophageal reflux disease. These associated factors increase mortality^1^^-^ 5. Reaches 600 million people worldwide and 30 million in Brazil. Including the population with overweight, the figure rises to 1.9 billion people worldwide and 95 million Brazilians^3^. Obesity is defined according to body mass index (BMI) greater or equal to 30^1^^-^ 3.

 Treatment is multidisciplinary and includes dietary measures, behavioral, exercise, medications, endoscopic and surgical methods^1^^,^ 2 ^,^ 6. Surgical treatment is consistent feature in severe obesity (IMC≥40 or ≥35 associated with comorbidities) with clinical treatment failure, reducing mortality rates and improving clinical comorbidities^1^^,^ 4. Surgical techniques can be restrictive (adjustable gastric banding, sleeve gastrectomy), disabsortive (duodenal switch, Scopinaro operation) or mixed (Roux-en-Y gastric bypass)^6^.

In the preoperative of patients with bariatric surgical indication, as well as history and appropriate physical examination, laboratory tests are required, including upper endoscopy (EDA). Its use in preoperatively asymptomatic patients is controversial^4^^,^ 8 ^,^ 9; however, most studies and societies recommend it in all patients^4^^-^ 8 ^,^ 10 for identifying various diseases to be treated before surgery. It may also suggest modification of surgical technique to be employed and to contraindicate the operation^4^^-^ 8. Studies have shown endoscopic findings in 80% of asymptomatic patients^7^^,^ 8.

The most frequent endoscopic findings are hiatal hernia, gastritis, esophagitis, gastroduodenal ulcers and Barrett's esophagus^4^^,^ 7 ^,^ 8. The stomach is the most affected segment up about 80% of the cases^5^.

The prevalence of *Helicobacter pylori* infection in individuals with bariatric surgery indication in the literature ranges from 8,7%^5^ to 30-40%^4^^,^ 11 of cases. It is recommended its search and pre-operative treatment^4^, since it is associated with higher incidence of gastric cancer^12^ and anastomotic mouth ulcers^4^.

The aim of this study was to analyze the EDA results in patients who were in pre-bariatric surgery, and underwent the procedure at the Endoscopy Unit of the Santa Casa de São Paulo, São Paulo, SP, Brazil, and compare them to findings in control group.

## METHOD

This study was approved by the Ethics Committee of the Santa Casa de São Paulo. The subjects involved were in agreement and consented to participate in the research and dissemination of its results in accordance with Resolution 196/96 of the National Health Council.

## Patients

The sample consisted of two groups, one being the control group. Were analyzed 35 patients in the group of obese in the preoperative period for bariatric surgery and 30 non-obese in the control group. The number of cases was calculated to obtain sample force power to 80% and significance level of 5% (p=0.05). All patients underwent endoscopy during the period from February to July 2014.

Were included in the study group those patients appointment to preoperative bariatric surgery survey. Were excluded those who refused to participate.

The control group was formed by a pairing of patients according to gender, age and use of proton pump inhibitors (PPIs). The age of the control group was established by calculating the average age range of bariatric patients, using 95% confidence interval.

Were included patients in the control group that had indication for EDA and with lower BMI than or equal to 29.9, being normal (BMI: 18.5 to 24.9) or overweight (BMI 25 to 29.9). Were excluded obese patients (BMI≥30) and the ones with gastrointestinal tract malignancy, stenosis, having prior gastrointestinal surgery or refused to participate. 

The variables analyzed were age, BMI, use of PPIs, symptoms, endoscopic findings, complications of the procedure, prevalence of infection of *Helicobacter pylori.*

## Endoscopy

Patients underwent to a questionnaire (protocol), followed by the completion of the endoscopic examination with standard 9.8 mm videoendoscope under sedation and topical anesthesia. The research for *Helicobacter pylori* was done by two methods: pathology and urease test, given as positive if any one of them was positive. Endoscopic findings were divided into ulceropeptic disease - gastritis, bulboduodenitis and peptic ulcers -; associated with gastroesophageal reflux disease - esophagitis, hiatal hernia, Barrett's esophagus -; polyps; others (diverticula, gastric intestinal metaplasia, etc.)

## Statistical analysis

For the organization of the data was used the spreadsheet MS-Excel version of MS-Office 2010, and to achieve the results was used IBM SPSS (Statistical Package for Social Sciences), version 22.0. The qualitative variables were represented by absolute frequency (n) and relative (%) and quantitative by average, standard deviation and median (md). Applying the Spearman, correlation analysis was performed in order to verify the degree of relationship between some of the variables. The application of Fisher's exact test was performed to verify possible differences between both groups for the variables of interest. The correlation coefficient (r) between the variables was determined as positive or negative. The significance level (p) was considered as less than 5% (p<0.05).

## RESULTS

The average age of the group of patients was 43.54 years (25-64) and the control group of 40.53 years (38-44) (Table 1).

**TABLE 1:** - Distribution of patients and the control group according to age and BMI

**Variable**	**Group**	**n**	**Mean**	**Standarddeviation**	**Minimum**	**Maximum**	**Percentile 25**	**Percentile 50 (median)**	**Percentile 75**
Age	Patient	35	43,54	10,94	25,00	64,00	36,00	42,00	54,00
	Control	30	40,53	1,70	38,00	44,00	39,00	40,50	42,00
IMC	Patient	35	47,26	6,21	38,00	68,00	43,10	45,90	49,50
	Control	30	24,21	1,98	21,00	28,00	22,68	23,80	25,90

Most individuals of both groups, patients and control, were women in 91.4% and 83.3%, respectively (Table 2).

**TABLE 2:** - Distribution of patient and the control groups by categorical variables

**Variable**	**Category**	**Group**			
		**Patient**	**Control**		
		**Freq.**	**Perc.**	**Freq.**	**Perc.**
Gender	F	32	91,40%	25	83,30%
	M	3	8,60%	5	16,70%
BMI	Normal	0	0,00%	21	70,00%
	Overweight	0	0,00%	9	30,00%
PPI user	Yes	5	14,30%	16	53,30%
	No	30	85,70%	14	46,70%
Symptomatic	Yes	3	8,60%	24	80,00%
	No	32	91,40%	6	20,00%
Pyrosis	Yes	2	5,70%	8	26,70%
	No	33	94,30%	22	73,30%
Regurgitation	Yes	1	2,90%	7	23,30%
	No	34	97,10%	23	76,70%
Epigastralgia	Yes	1	2,90%	18	60,00%
	No	34	97,10%	12	40,00%
Other symptom	Yes	1	2,90%	1	3,30%
	No	34	97,10%	29	96,70%
Normal	Yes	7	20,00%	10	33,30%
	No	28	80,00%	20	66,70%
Endoscopic finding - DUP	Yes	20	57,10%	15	50,00%
	No	15	42,90%	15	50,00%
Endoscopic finding - DRGE	Yes	12	34,30%	7	23,30%
	No	23	65,70%	23	76,70%
Endoscopic finding - polyp	Yes	4	11,40%	2	6,70%
	No	31	88,60%	28	93,30%
Endoscopic finding - others	Yes	3	8,60%	2	6,70%
	No	32	91,40%	28	93,30%
Helicobacter pylori	Positive	21	60,00%	12	40,00%
	Negative	14	40,00%	18	60,00%

PPI=proton pump inhibitor; DUP=ulceropeptic disease; DRGE=reflux disease

The average value of BMI in the group of patients was 47.26 kg/m^2^ (38-68) and in the control group of 24.21 kg/m^2^ (21-28) (Table 1). Only one individual of patient group had BMI below 40 kg/m^2^. Most of the control group was of normal individuals (70%).

The ones analyzed in the group of patients, 30 (85.7%) did not use PPIs and five (14.3%) yes. Sixteen of control group (53.3%) used PPIs and 14 did not (Table 2).

Most patients were asymptomatic (91.4%); in the three symptomatic the most prevalent symptom was heartburn. Most control subjects were symptomatic (80%). The most prevalent symptom was epigastric pain.

Twenty-eight (80%) patients had endoscopy with alterations and seven (20%), normal. In the control group ten (33.3%) had normal results and 20 (66.7%) amended (Table 2). Twenty-six (81.25%) of the 32 asymptomatic patients had endoscopy with alterations.

**FIGURE 1:**
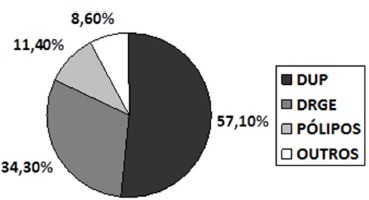
- Endoscopic findings in patients group

The endoscopic changes in the patient group were 57.1% (n=20) resulting from ulceropeptic disease, 34.3% (n=12) associated with reflux disease, 11.4% (n=4) showed benign polyps and 8.6% (n=3) other findings - Zenker's diverticulum, esophageal and gastric intestinal metaplasia, subepithelial lesions (Figure 1, Table 2)

DUP= ulceropeptic disease; DRGE=reflux disease

**FIGURE 2:**
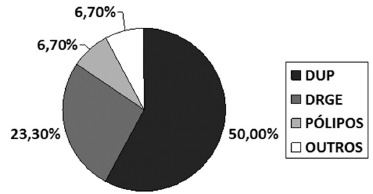
- Endoscopic findings in control group

In the group of patients, the analysis of correlation between the increase in the value of BMI and the incidence of endoscopic findings was not statistically significant (Table 3). In the control group, the endoscopic findings were 50% (n=15) resulting from ulceropeptic disease, 23.3% (n=7) associated with reflux disease, two had benign polyps and two other findings, which were ectopic pancreas and ectopic gastric mucosa in proximal esophagus (Figure 2; Table 2).

**TABLE 3:** - Correlation analysis between the increase in the value of BMI and the incidence of endoscopic findings

**Variable**	**Statistic**	**IMC**
DUP	Correlation coefficient (r)	+0,031
	Calculated significance (p)	0,858
	n	35
DRGE	Correlation coefficient (r)	-0,271
	Calculated significance (p)	0,115
	n	35
Polyp	Correlation coefficient (r)	+0,013
	Calculated significance (p)	0,939
	n	35
Others	Correlation coefficient (r)	+0,207
	Calculated significance (p)	0,232
	n	35

DUP= ulceropeptic disease; DRGE=reflux disease

The analysis of the number of endoscopic findings in patients and in the control group did not show statistically significant differences (Table 4).

DUP= ulceropeptic disease; DRGE=reflux disease

**TABLE 4:** - Analysis of endoscopic findings of the patients group compared to the control group

**Variable**	**Category**	**Group**	**p**			
		**Patient**	**Control**			
		**Freq.**	**Perc.**	**Freq.**	**Perc.**	
DUP	Yes	20	57,10%	15	50,00%	0,565
	No	15	42,90%	15	50,00%	
DRGE	Yes	12	34,30%	7	23,30%	0,333
	No	23	65,70%	23	76,70%	
Polyp	Yes	4	11,40%	2	6,70%	0,508
	No	31	88,60%	28	93,30%	
Others	Yes	3	8,60%	2	6,70%	0,774
	No	32	91,40%	28	93,30%	

DUP= ulceropeptic disease; DRGE=reflux disease

The prevalence of *Helicobacter pylori* infection was 60% (n=21) individuals in the group of patients and in 40% (n=12) in the control group (Table 2).

## DISCUSSION

In this study, 97.1% (n=34) of patients preoperatively showed IMC≥40, being included, therefore, in a group of severely obese, whose surgical treatment may bring good results.

There are studies showing that most individuals in the preoperative bariatric surgery does not have symptoms of reflux^5^. In this study, the majority of individuals in the group of patients were asymptomatic (91.4%) and 30 (85.7%) did not use PPIs. The most common symptoms were heartburn and epigastric pain between symptomatic individuals from groups of patients and control.

There is published data that showed endoscopic findings in patients in the preoperative bariatric surgery ranging between 9.5% and 90%, most of them between 18% and 54%^9^.

In this study, 81.25% (n=26) of asymptomatic individuals in the group of patients had alterations in endoscopic examination, confirming some studies showing endoscopic findings in 80% of asymptomatic patients^7^^,^ 8.

Endoscopic changes in the patient group were 57.1% (n=20) resulting from ulceropeptic disease and 34.3% (n=12) associated with reflux disease, corroborating data in the literature which show that the most frequent endoscopic findings are gastritis, hiatal hernia, esophagitis and gastroduodenal ulcers^4^^,^ 7 ^,^ 8. Individuals analyzed in this study had no endoscopic finding that could contraindicate bariatric surgery.

In the group of patients, the analysis of correlation between the increase in the value of BMI and the incidence of endoscopic findings was not statistically significant, suggesting that there is no relationship between the degree of obesity and the occurrence of endoscopic changes.

The analysis of the number of endoscopic findings in patients in the control group did not show statistically significant differences, which may suggest that obese individuals have no higher probability of having endoscopic changes.

The prevalence of *Helicobacter pylori* in this study was 60% (n=21) in the group of patients. In the literature, its prevalence in individuals with bariatric surgery indication varies from 8,7%^5^ a 30-40%^4^^,^ 11. The finding of this study may have been due to the use of two methods for the bacteria search (urease test and histology), which increased the accuracy. One should also take into account that there are papers that used only one diagnostic method and others investigated*Helicobacter pylori* only in part of their series. However, it is recommended to look for and treat it in the pre-operative period, since it is associated with higher incidence of gastric cancer^12^ and mouth ulcers in the anastomoses^4^.

## CONCLUSION

It is recommended that endoscopy should be performed in all patients in the preoperative of bariatric surgery, although the degree of obesity is unrelated to larger endoscopic changes. The obese do not have more endoscopic changes than the non-obese.
